# MspA Nanopores from Subunit Dimers

**DOI:** 10.1371/journal.pone.0038726

**Published:** 2012-06-18

**Authors:** Mikhail Pavlenok, Ian M. Derrington, Jens H. Gundlach, Michael Niederweis

**Affiliations:** 1 Department of Microbiology, University of Alabama at Birmingham, Birmingham, Alabama, United States of America; 2 Department of Physics, University of Washington, Seattle, Washington, United States of America; Université d’Evry val d’Essonne, France

## Abstract

*Mycobacterium smegmatis* porin A (MspA) forms an octameric channel and represents the founding member of a new family of pore proteins. Control of subunit stoichiometry is important to tailor MspA for nanotechnological applications. In this study, two MspA monomers were connected by linkers ranging from 17 to 62 amino acids in length. The oligomeric pore proteins were purified from *M. smegmatis* and were shown to form functional channels in lipid bilayer experiments. These results indicated that the peptide linkers did not prohibit correct folding and localization of MspA. However, expression levels were reduced by 10-fold compared to wild-type MspA. MspA is ideal for nanopore sequencing due to its unique pore geometry and its robustness. To assess the usefulness of MspA made from dimeric subunits for DNA sequencing, we linked two M1-MspA monomers, whose constriction zones were modified to enable DNA translocation. Lipid bilayer experiments demonstrated that this construct also formed functional channels. Voltage gating of MspA pores made from M1 monomers and M1-M1 dimers was identical indicating similar structural and dynamic channel properties. Glucose uptake in *M. smegmatis* cells lacking porins was restored by expressing the dimeric *mspA M1* gene indicating correct folding and localization of M1-M1 pores in their native membrane. Single-stranded DNA hairpins produced identical ionic current blockades in pores made from monomers and subunit dimers demonstrating that M1-M1 pores are suitable for DNA sequencing. This study provides the proof of principle that production of single-chain MspA pores in *M. smegmatis* is feasible and paves the way for generating MspA pores with altered stoichiometries. Subunit dimers enable better control of the chemical and physical properties of the constriction zone of MspA. This approach will be valuable both in understanding transport across the outer membrane in mycobacteria and in tailoring MspA for nanopore sequencing of DNA.

## Introduction

Pore proteins have important transport functions in biological cells [1], [2] and are versatile tools in nanotechnology which have been utilized as sensors of small molecules [3], proteins [4] and nucleic acids [5]. Most of these technological applications have been pioneered with α-hemolysin from *Staphylococcus aureus*. The α-hemolysin pore is a 14-stranded β-barrel composed of seven subunits. The membrane-spanning β-barrel has a length of 5 nm. It has a rather broad constriction zone consisting of 3 amino acids with a diameter of ≈1.5 nm [6].

MspA is a homo-octameric channel-forming protein which provides the major diffusion pathway of *Mycobacterium smegmatis* for hydrophilic solutes [7], [8], [9]. The goblet-like structure of MspA has a single short (∼0.5 nm) constriction consisting of two amino acids (D90, D91) with a diameter of ≈1 nm [7]. In addition, MspA is very robust: it retains its octameric structure after exposure to solutions of pH 0 to 14, to 100°C for 30 min, and prolonged exposure to strong denaturing agents such as SDS [10]. Thus, owing to its tremendous stability and its pore geometry MspA is better suited as a sensing device in nanotechnological applications such as DNA sequencing where, compared to α-hemolysin, larger signal-to-noise ratios of current fluctuations through the short constriction zone of MspA were observed [11].

In addition, MspA is the prototype of the MspA superfamily (Accession: cl07724) in the Conserved Domain Database [12] comprising more than 60 channel proteins from different *Mycobacterium* species and other *Corynebacterineae* such as *Rhodococcus, Segniliparus and Gordonia*, all of which are uncharacterized except MspA. Notably, MspA is absent in slow growing mycobacteria such as *M. tuberculosis* and in corynebacteria such as *Corynebacterium glutamicum*. The octameric symmetry as the predominant structure is a novel feature of channel-forming proteins. The protective antigen (PA) of *Bacillus anthracis* forms a heptameric channel with a β-barrel transmembrane motif [13] similar to that of the staphylococcal α-hemolysin. However, it was recently observed that assembly of PA in the presence of its toxin ligands produces more stable, octameric PA channels [14]. A similar situation may exist for MspA where minor bands with altered electrophoretic mobilities are observed in preparations both from *M. smegmatis* and after refolding of recombinant MspA monomer from *E. coli* [7], [15].

**Figure 1:**
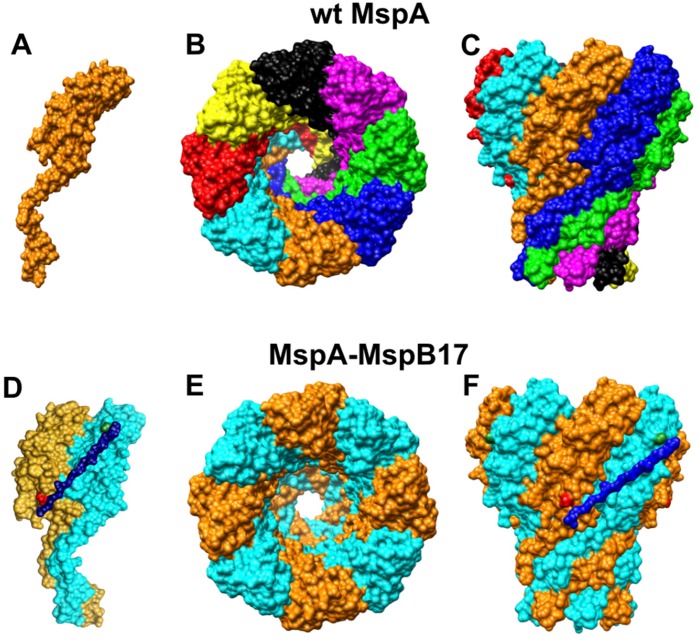
Schematic representation of MspA and MspA-MspB subunit dimer MspA monomer (A) is encoded by a single copy of *mspA* gene. Eight monomers self assemble in the outer membrane of *Mycobacterium smegmatis* to form a functional pore (B and C, top and side view, respectively). MspA-MspB dimer (D) is connected by a (GGGGS)_3_ linker via C-terminal asparagine of MspA subunit (shown in red) and N-terminal glycine (in green) of MspB subunit. MspA-MspB dimer (E and F, top and side view, respectively) form a channel in the outer membrane of *M. smegmatis.*

To examine whether MspA can indeed form channels with different symmetries, we chose to control the stoichiometry of the MspA pore by producing concatenated subunits. Such an approach was recently used for α-hemolysin [16] and should be feasible for MspA as well, since the N- and the C-termini of neighbouring MspA monomers are close together (Fig. 1). However, it was unclear whether the structure of MspA [7] and its particular membrane topology with both termini presumably buried in the outer membrane of *M. smegmatis* [17] is compatible with formation of a single-chain MspA pore.

In this study we constructed concatenated MspA dimers which resulted in the formation of tetrameric pores. We present evidence that these single-chain dimers assemble to channels in the outer membrane of *M. smegmatis* with very similar properties compared to wt MspA pores indicating that the overall number of eight MspA subunits was not altered in the engineered MspA pore. These results represent a key step in altering the subunit assembly of MspA to increase the control of the chemical and biophysical properties of the MspA channel for nanotechnological applications such as DNA sequencing. By using MspA composed of concatenated dimers we significantly reduce the formation of hetero-oligomers and avoid random self-assembly of mutated and native subunits during *msp* gene expression in *M. smegmatis*. Overall, these results open new avenues to specifically adapt the MspA pore to requirements in basic science and nanotechnology.

## Results

### Influence of the Linker on Expression Levels of Pores Made of Msp Dimers in *M. smegmatis*

The distance between the N- and C-termini of two neighbouring MspA monomers in the crystal structure is 4.5 nm [7] and could be bridged by a peptide of at least 15 amino acids to form a subunit dimer (Fig. 1D). Membrane topology experiments indicated that both N- and C-termini of adjacent MspA monomers are buried in the outer membrane of *M. smegmatis* [17]. Thus, a putative linker connecting the termini might be exposed to the membrane imposing constraints on the linker sequence. Hence, in a first step, we examined different linker lengths and sequences and examined their effects on *mspA* expression and channel formation by the MspA pore.

In order to avoid recombination between two identical *mspA* genes in a fusion construct, we chose to connect the *mspA* gene with the *mspB* gene which encodes a porin with only two amino acid substitutions compared to MspA: alanine 138 is located in the extracellular loop L9, while glutamate 139 is the first residue of β-sheet β−10 in the rim domain of the MspA pore. Both residues do not participate in subunit interactions in the MspA octamer [7]. These *msp* genes differ by 7% [8] reducing the likelihood of homologous recombination between the neighboring genes on the expression plasmid significantly.

**Figure 2:**
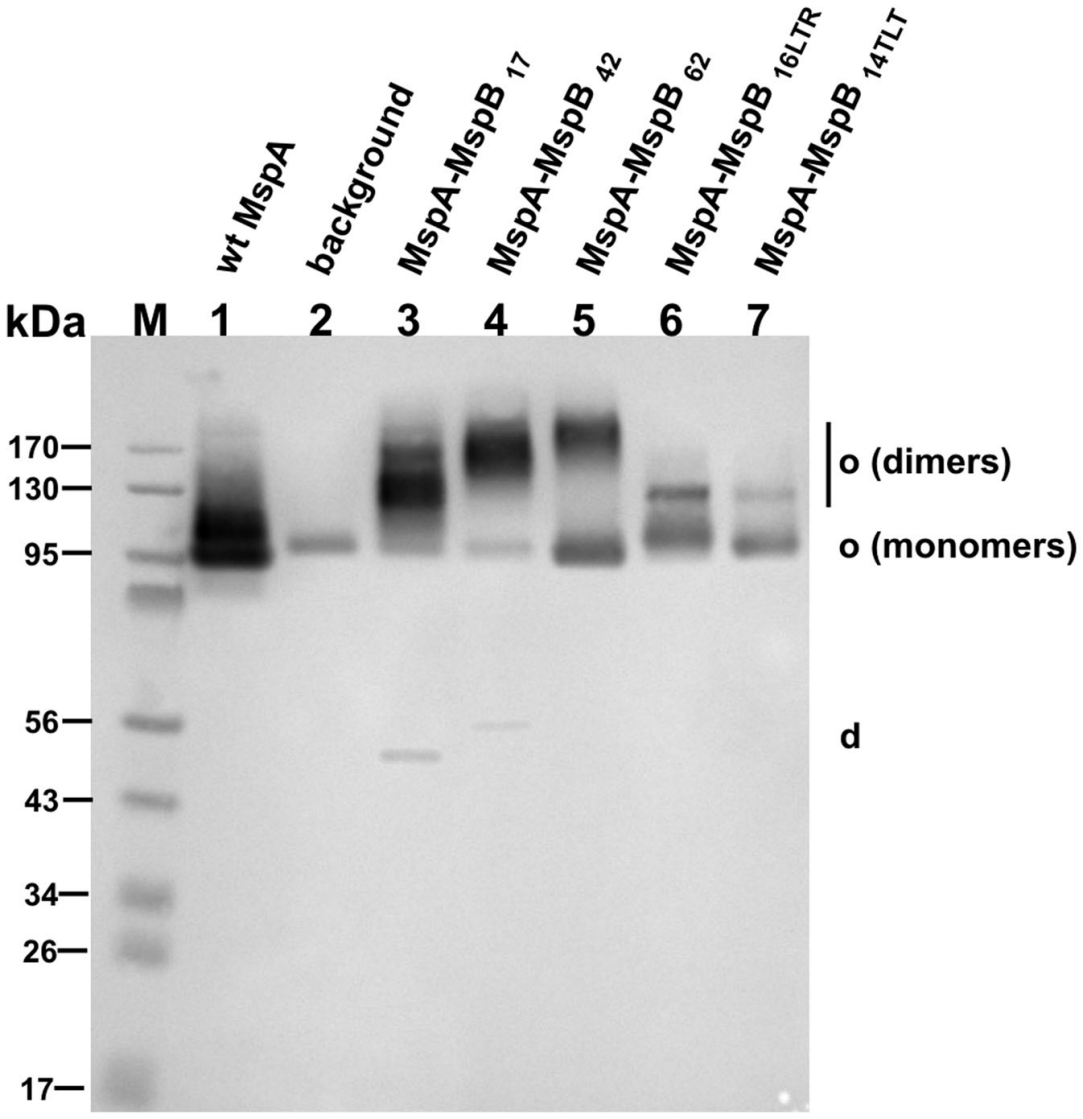
Expression of *mspA-mspB* fusions in *M. smegmatis.* Western blot of detergent extracts of the porin mutant *M. smegmatis* ML16 expressing different MspA constructs. 15 µl of the extracts were loaded onto 10% polyacrylamide gel followed by transfer onto a PVDF membrane and detection with a polyclonal MspA antiserum. Lanes: M, molecular mass marker; 1, pMN016 (wt *mspA*); 2, empty vector pMS2; 3, pML870 (*mspA-mspB_17_*); 4, pML870-10 (*mspA-mspB_42_*); 5, pML870-6 (*mspA-mspB_62_*); 6, pML871 (*mspA-mspB_16LTR_*); 7, pML872 (*mspA-mspB_14TLT_*). Abbreviations: o, oligomeris form; d, dimeric form.

Initially, we tested a glycine/serine peptide as a linker because it is flexible and does not appear to induce conformational changes of connected subunits [18]. To identify a suitable linker length we constructed several MspA-MspB subunit dimers connected by 17, 42, and 62 amino acid long peptides (Table 1). The genes encoding the different *mspA-mspB* fusions were expressed from plasmids (Table 1) in the porin triple mutant *M. smegmatis* ML16, which lacks the *mspA*, *mspC*, and *mspD* genes. The amount of endogenous porins in the outer membrane of this strain is reduced by 15-fold [19]. The MspA proteins were extracted from whole cells of *M. smegmatis* using a selective extraction with 0.5% n-octyl-POE at 100°C as described earlier [10]. A Western blot probed with MspA antibodies showed that all fusion proteins gave rise to heat-stable oligomeric proteins (Fig. 2). The apparent size of these oligomers increased with increasing linker lengths indicating that these proteins were made from linked subunit dimers.

**Table 1:** MspA dimers tested in the study.

Construct name	Genes linked	Linker sequence	Linker length inamino acids	Plasmid*
MspA-MspB_17_	*mspA-mspB*	DI(GGGGS)_3_	17	pML870
MspA-MspB_42_	*mspA-mspB*	DI(GGGGS)_8_	42	pML870-10
MspA-MspB_62_	*mspA-mspB*	DI(GGGGS)_12_	62	pML870-6
MspA-MspB_16LTR_	*mspA-mspB*	LTREWFHSGRAKYIVA – 38–51 residues ofwtMspA	16	pML871
MspA-MspB_14TLT_	*mspA-mspB*	TLTVQQWDTFLNGV – 15–29 residues ofwtMspA	14	pML872
M1-M1_19_	*mspA_M1_-mspA_M1_*	GT(GGGGS)_3_MH	19	pML2632

* - all constructs were expressed in *M. smegmatis* ML16.

However, the amount of oligomers consisting of unlinked monomers also increased with the length of the linker peptide (Fig. 2, lanes 3–5). For example, 92% of the MspA-MspB_17_ protein was observed in oligomeric form, while approximately half (48%) of MspA-MspB_62_ appeared to be an oligomer composed of monomeric subunits (Fig. 2, lane 5). These results were consistent with denaturation experiments (see Experimental procedures), which yielded predominantly monomers for wt MspA and dimers for the MspA-MspB_17_ and MspA-MspB_42_ fusion proteins (Fig. S1). By contrast, approximately half of the MspA-MspB_62_ fusion protein resulted in formation of dimers, while the other half yielded monomers after denaturation (Fig. S1) indicating a truncation of the protein, probably due to an increased accessibility of the long linker to cellular proteases.

Since ssurface accessibility experiments with a membrane-impermeable biotinylation reagent indicated that the N- and C- termini of an MspA monomer are embedded in the outer membrane of *M. smegmatis* [17], we also tested linker sequences which are more hydrophobic than a glycine/serine peptide. We chose two peptides whose sequences are identical to the membrane-embedded β strands 2 and 3 (plus loop 2) of MspA (Table 1). Changing the linker sequence from glycine/serine to a sequence with alternating hydrophilic and hydrophobic amino acids (MspA-MspB_16LTR_, MspA-MspB_14TLT_) reduced the overall level of MspA oligomers and in particular the amount of oligomers made from subunit dimers (Fig. 2).

Next, we quantified expression levels of the different MspA-MspB subunit dimer constructs. When whole cells of *M. smegmatis* ML16 with an *mspA* expression plasmid were treated with octyl-POE only MspA oligomers were observed (Fig. S2, A). A negligible amount of MspB was detected in extracts of *M. smegmatis* ML16 carrying an empty vector (Fig. S2A), indicating a very low background expression of the *mspB* gene in this strain. All MspA-MspB subunit dimers had reduced expression levels compared to wt MspA (Fig. S2, A). Quantitative image analysis of the bands corresponding to octameric protein showed that MspA-MspB_17_ and MspA-MspB_42_ constructs had expression levels of 9%, and 11% of that of wt, respectively (Fig. S2, B). Expression of MspA-MspB_62_ (60% of wt) was higher because of the formation of a truncated fusion protein. Quantitative image analysis of a Western blot (Fig. S1) indicated that 53% of the MspA-MspB_62_ consisted of truncated monomers. Taken together, these results show that the amount of MspA oligomers made from subunit dimers was the highest for the shortest linker sequence (17 amino acids).

### Analysis of Channel Activity of MspA-MspB Dimers in Lipid Bilayers

Lipid bilayer conductance measurements provide direct evidence whether a particular protein forms functional channels in lipid membranes [20]. Moreover, single-channel measurements are indicative of the structure and stoichiometry of the channel [21]. In order to examine the channel activity of MspA-MspB dimers, the genes encoding MspA and MspB or the different Msp dimers (Table 1) were expressed in the porin mutant *M. smegmatis* ML16, extracted using the detergent n-octyl-polyoxyethylene and purified by anion exchange and gel filtration chromatography as described [10].

Since the dimeric subunit of these fusion proteins consisted of MspA and MspB, we first analyzed the single channel conductance of MspA and MspB in order to establish reference conductance values. No pores were recorded in control experiments when only detergent-containing buffer was added to the lipid bilayer (not shown). Both wt MspA and wt MspB rapidly inserted into the bilayer after addition to the cuvette, resulting in a step-wise increase of the ionic current (Fig. S3). In agreement with our previous results, analysis of the conductance of the wt MspA showed a major peak at 4.9 nS (Fig. S3, B). Interestingly, analysis of wt MspB showed a bimodal distribution of conductance with peaks at 2.4 nS and 3.4 nS (Fig. S3, D). The reduced conductance of wt MspB is surprising, given the fact that MspB differs from MspA only in two residues which are located in the rim domain of the pore and, hence, are not likely to contribute to the ion flow.

**Figure 3:**
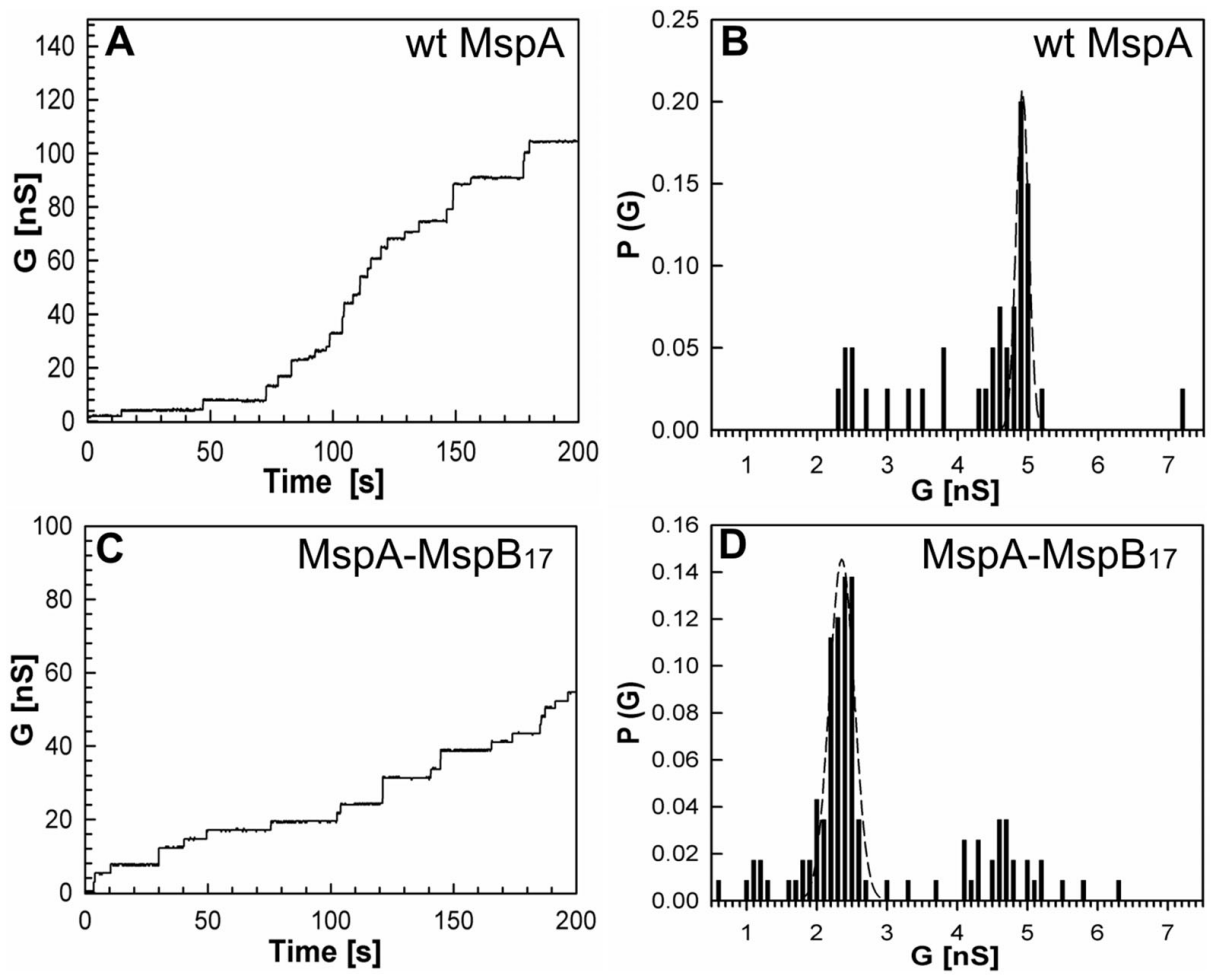
Single-channel recordings and analysis of conductance of purified MspA and MspA-MspB_17_ dimer in lipid bilayer. Single-channel recordings of purified wt MspA (A) and MspA-MspB_17_ dimer (C) in a diphytanoyl phosphatidylcholine (DphPC) membrane in the presence of approximately 100 ng/mL protein sample. Protein solutions were added to both sides of the membrane and data were collected from at least five different membranes. −10 mV transmembrane potential was applied and current was measured in 1 M KCl solution, pH 7.0 Analysis of single-channel conductances of wtMspA (B) and MspA-MspB_17_ dimer (D). To avoid possible contamination of the MspA-MspB_17_ preparation with MspB, the subunit dimer protein was excised from the gel and electro-eluted. Analysis of the probability P of a conductance step G for single-channel events. The average single-channel conductances were 4.8 nS, and 2.2 nS for wt MspA, and MspA-MspB_17_ dimer, respectively.

Next, we analyzed the channel forming properties of MspA-MspB fusion proteins (Table 1). Step-wise increase in current in lipid bilayer experiments was observed when MspA-MspB_17_ was added to the cuvette. Analysis of the single channel conductances showed that MspA-MspB_17_ had a major peak at 2.3 nS (Fig. 3, D), similar to the MspB pore (Fig. S3, D). The observation that the channel activity of the purified pores containing the MspA-MspB dimer construct was much higher than extracts from *M. smegmatis* ML16 (background) indicated that oligomers containing the MspA-MspB dimer form pores. To exclude that the observed activity in these protein samples was caused by minor contaminations with MspB which is present in *M. smegmatis* ML16 on a low level, the protein band representing oligomeric Msp made from dimers (see Fig. 2) was excised from the gel and the protein was extracted from polyacrylamide. This protein also formed channels in lipid bilayer experiments demonstrating that the oligomer made from MspA-MspB dimers is indeed a functional pore. This result further shows that the Msp proteins purified by chromatographic methods consists mainly of oligomers made from MspA-MspB dimers. It is concluded that the (GGGGS)_3_ linker does not compromise the channel activity of the Msp pore (Fig. 3D).

Pore proteins purified from *M. smegmatis* ML16 carrying expression plasmids encoding MspA-MspB_42_, MspA-MspB_62_, MspA-MspB_16LTR_, and MspA-MspB_14TLT_ also resulted in a step-wise increase in current, suggesting these proteins formed functional channels (Fig. S4, C, E, G, I). Analysis of the conductances showed that MspA-MspB_42_, MspA-MspB_62_, and MspA-MspB_LTR_ dimers had major conductance peaks at 2.3 nS (Fig. S4, D, F, H). MspA-MspB_14TLT_ did not show a uniform peak. In addition to 2.3 nS the analysis showed two extra peaks at 0.9 nS and 4.9 nS (Fig. S4, J). It should be noted, that given a significant amount of truncated fusion subunit in these samples, the octameric form of these channels may not be solely composed of fused subunit dimers. For example, an octamer made from an MspA-MspB fusion protein may be predominantly composed of truncated MspB units. This would give rise to a more complex conductance distribution pattern as observed for MspA-MspB_14TLT_. Notably, channel activity of all dimeric constructs was reduced in comparison to wt MspA as the number of insertions per time was decreased for all dimers as evident from Fig. S4. This is consistent with lower protein concentrations in the detergent extract (Fig. S2) used in the lipid bilayer experiments. Taken together, the lipid bilayer experiments showed that MspA-MspB dimers formed functional channels.

### Construction, Purification, and Expression of M1-M1_19_ MspA in M. smegmatis ML16

**Figure 4:**
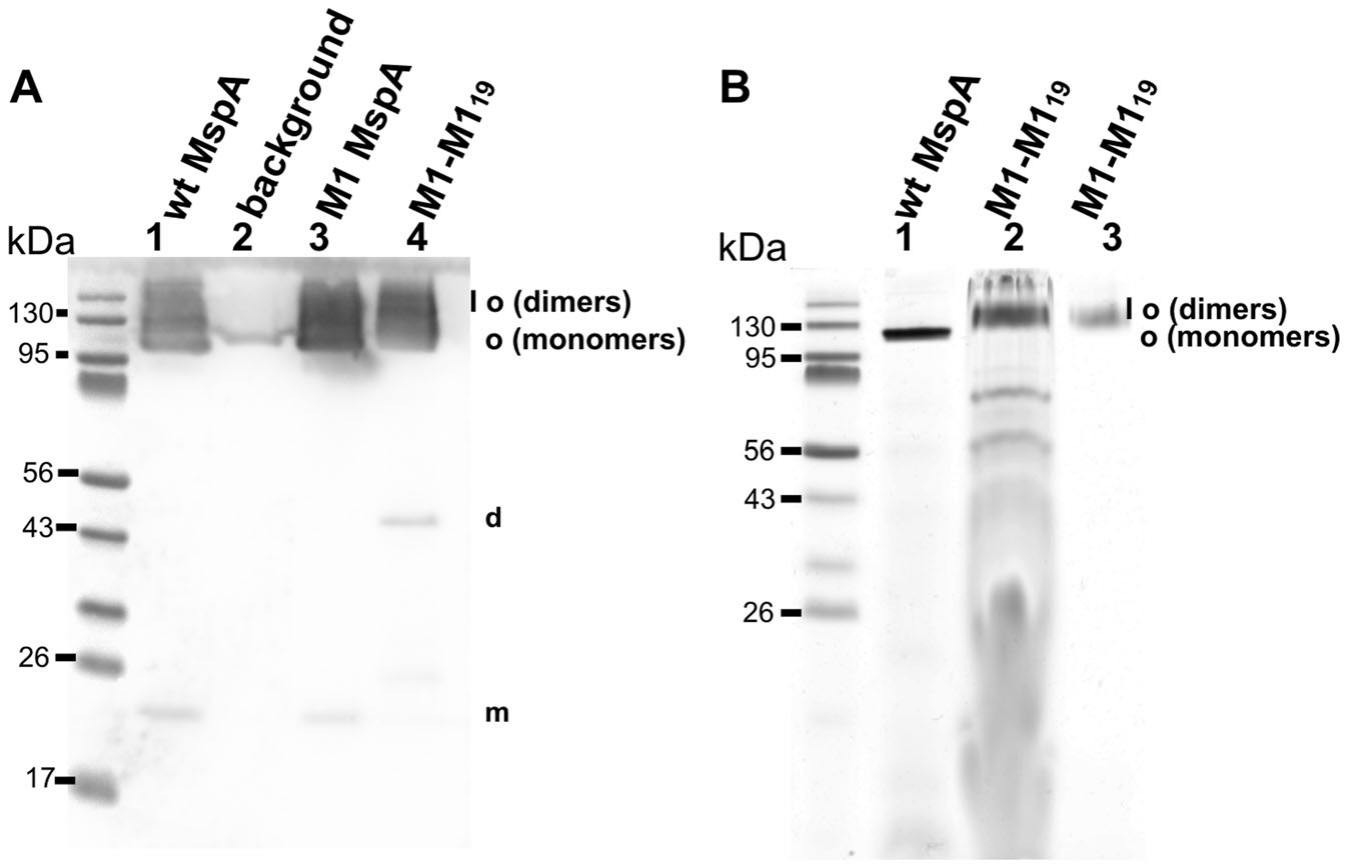
Expression of M1-M1_19_ MspA on the surface of *M. smegmatis* and purification by gel extraction. (A) Western blot of the samples after selective extraction of MspA proteins at 100°C from *M. smegmatis* ML16 cells. 15 µl of the extracts have been loaded onto 10% polyacrylamide gel followed by transfer onto the PVDF membrane, and detected with polyclonal MspA antiserum. Lanes: 1, raw extract of MspA; 2, empty vector pMS2; 3, M1 MspA; 4, M1-M1 MspA. Molecular mass marker is shown next to the lane 1. (B) Analysis of M1-M1_19_ MspA gel extraction procedure. After selective extraction of M1-M1_19_ MspA from *M. smegmatis* ML16 cells the sample was concentrated and loaded onto SDS-PAGE. The band corresponding to oligomeric form of the protein was cut from the gel and electoreluted followed by analysis for channel forming properties. After bilayer experiments the sample was loaded on the gel to monitor its stability. Lanes: 1, raw extract of wt MspA; 2, M1-M1_19_ MspA before gel extraction and bilayer analysis; 3, M1-M1_19_ after gel extraction and bilayer analysis. Molecular mass marker is shown next to lane 1. SDS-PAGE gel was stained with Simple Blue Safe Stain (Invitrogen). Abbreviations: o, oligomeris form; d, dimeric form; m, monomeric form.

The MspA mutant D90N/D91N/D93N (M1) has been shown to translocate ssDNA and has the ability to resolve single-nucleotides in single-stranded DNA [11], [21]. In order to utilize the improved control over the design of the constriction zone of MspA for nanosequencing of DNA, we constructed the M1-M1 MspA dimer. To this end we chose the (GGGGS)_3_ linker because the MspA-MspB_17_ dimer showed stability similar to the octameric pore and had highest channel activity of all tested pores made from MspA-MspB dimers. A gene containing a *mspA M1-mspA M1* translational fusion connected by a linker sequence encoding for (GGGGS)_3_ was synthesized and cloned into an expression plasmid (pML2632). To check whether M1-M1_19_ MspA was expressed, Msp proteins were extracted by heating the whole cells in the presence of 0.5% n-octyl-POE. Figure 4 shows that the M1-M1_19_ subunit dimer is expressed and stable in its oligomeric form even in a denaturing protein gel. However, the expression levels of the M1-M1_19_ MspA subunit dimer in *M. smegmatis* ML16 was reduced by 12-fold in comparison to wt MspA or M1 MspA similar to most other MspA dimers (Fig S2). It should be noted that the two additional amino acid residues in the linker sequence of M1-M1_19_ subunit dimer had no effect on expression in *M. smegmatis*. Both MspA-MspB_17_ and M1-M1_19_ had similar expression levels of 9% and 8% of the wt level, respectively (Fig. S2, B). Purification of M1-M1_19_ MspA from *M. smegmatis* ML16 (Fig. S5) using our standard protocol [10], [22] yielded 15 µg per liter of culture of apparently pure M1-M1_19_ MspA. This is approximately one tenth of the yield of wt MspA consistent with the above estimate of protein levels in raw detergent extracts.

### Single Channel Activity of M1-M1_19_ MspA in Lipid Bilayers

**Figure 5:**
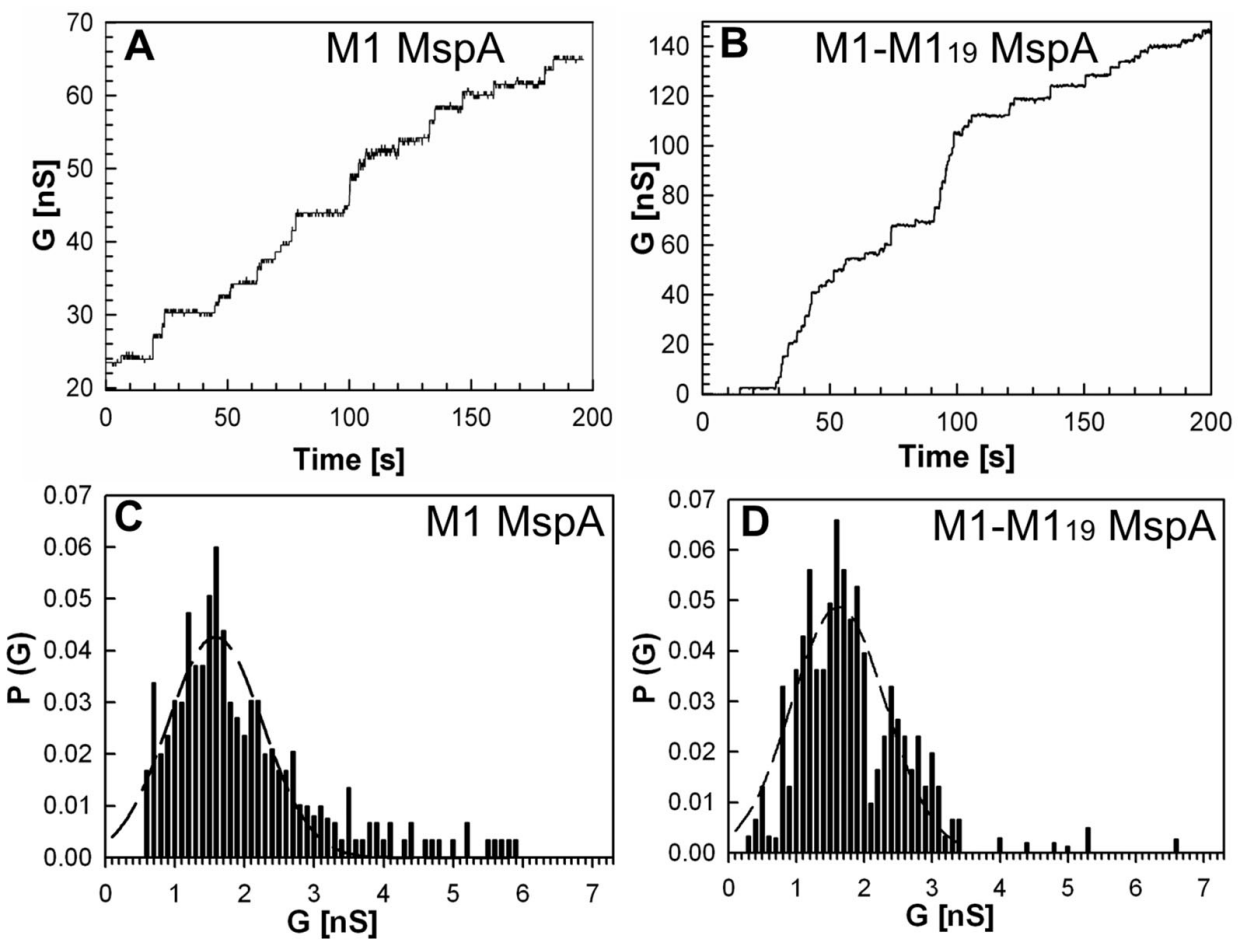
Characterization of M1 MspA and M1-M1_19_ MspA dimer in lipid bilayer experiments. Single-channel recordings of purified M1 MspA (A) and gel extracted M1-M1_19_ MspA (B) in a diphytanoyl phosphatidylcholine membrane in the presence of approximately 100 ng/mL protein. Protein solutions were added to both sides of the membrane and data were collected from at least five different membranes. −10 mV transmembrane potential was applied and current was measured in 1 M KCl solution, pH 7.0. Analysis of single-channel conductances of M1 MspA and M1-M1_19_ MspA dimer (C and D, respectively). Analysis of the probability P of a conductance step G for single-channel events. The peak single-channel conductance for both M1 MspA and gel extracted MspA made from M1-M1_19_ subunit dimer was 1.6 nS.

To determine whether M1-M1_19_ MspA forms a functional pore and to avoid contamination by truncated constructs or by MspB monomers, we separated MspA oligomers made from monomeric and dimeric subunits by gel electrophoresis and excised the M1-M1_19_ oligomer from the gel as described in Experimental Procedures. Figure 4B shows the purified M1-M1_19_ oligomer before and after the extraction procedure (lanes 2 and 3, respectively). The channel forming properties of oligomeric MspA made from M1 monomers and subunit dimers were examined by lipid bilayer experiments. No pores were recorded in control experiments when only detergent-containing buffer was added to the lipid bilayer (not shown). Addition of 100 ng/ml of either M1 MspA or gel-purified M1-M1_19_ MspA into the cuvette produced a step-wise increase in the current suggesting that both mutants formed open channels in lipid bilayer experiments (Fig. 5, A and B). The single-channel conductances and the channel activities of both proteins were very similar with a peak at 1.6 nS (Fig. 5, C and D) indicating that the covalent connection of the termini of neighbouring subunits did not change the channel properties of M1 MspA. This is consistent with our findings for MspA-MspB dimers.

### Voltage Gating of M1-M1_19_ MspA in Lipid Bilayers

Voltage gating is often observed for porins and reflects complex changes inside channels leading to pore closure in response to increased electrical field strength [23], [24]. This phenomenon is not well understood on a molecular level [25], [26], but could be utilized as an indicator of the overall structural and dynamic channel properties. To examine whether the linkage of two MspA subunits by a peptide influences the overall structure of the MspA pore we analyzed the voltage gating of M1-MspA and of M1-M1_19_ MspA. Upon insertion of approximately 100 pores into a lipid bilayer, the polarity of the membrane potential was reversed and the potential was increased stepwise in 10 mV increments. No channel closure of M1 MspA and M1-M1_19_ MspA was observed at voltages up to - 40 mV (Fig. S7). At −50 mV approximately 12% of M1 MspA and 15% of M1-M1_19_ MspA pores closed spontaneously. Similar to wt MspA, both channels stayed open at +20 mV potential (Fig. S7). However, membrane current decreased exponentially at +40 mV potential when either M1 MspA or M1-M1_19_ MspA were present in the lipid bilayer. These results show that the MspA pores made from M1 monomers and from M1-M1_19_ subunit dimers have very similar channel properties and indicate that the overall structure and channel properties of the MspA pore were not much altered by connecting two monomers by a small peptide linker.

### Analysis of the M1-M1_19_ MspA Dimer in Vivo

**Figure 6:**
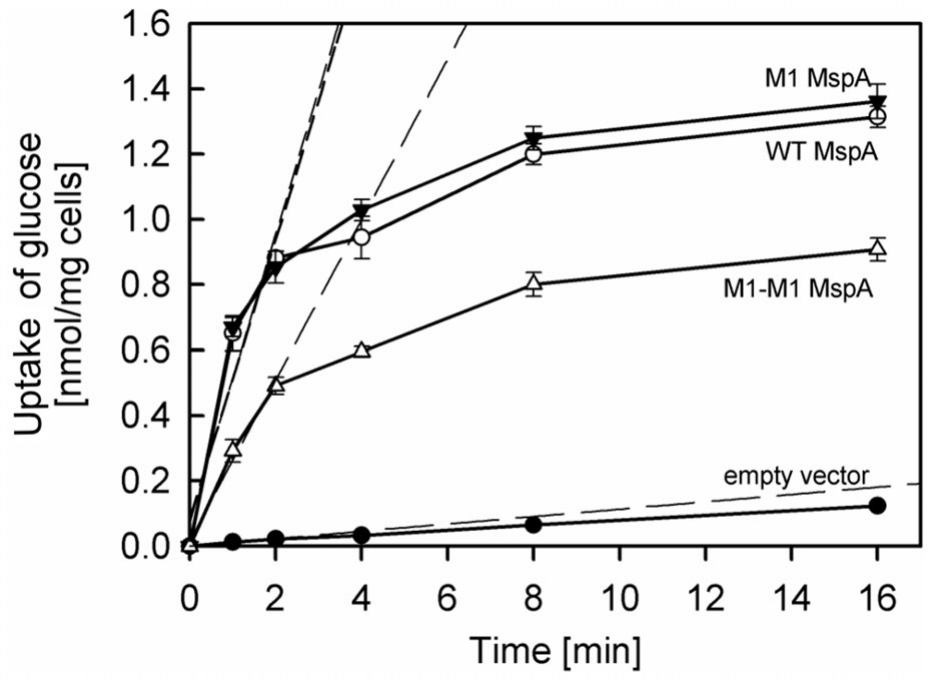
Uptake of glucose by *M. smegmatis* ML16. Accumulation of [^14^C]glucose by *M. smegmatis* ML16 expressing wt MspA, empty vector pMS2, M1 MspA, and M1-M1_19_ MspA was measured. The experiments were done in triplicates. The data are shown as averages ± standard deviations. The assay was performed at 37°C at a final glucose concentration of 1 µM. The cells were grown to an A_600_ ∼0.6. At indicated time points 200 µL of cells were drawn from a vial, applied on a 0.22 µm cellulose filter, washed several times with LiCl, and counted on a scintillation counter. Dashed lines represent regression analysis of the first three data points for each strain. Uptake rates for ML16 expressing wt MspA, empty vector, M1 MspA, and M1-M1_19_ MspA were 0.42, 0.01, 0.44, 0.24 nmol/mg cells/min, respectively.

Another indicator of gross structural properties of a porin is its correct folding and localization as a functional pore in its native membrane. To assess whether linking of two monomers to a dimer influences these properties of MspA, we examined the accumulation of glucose which was used as a reference solute for porin activity in *M. smegmatis* [8], [19], [27]. *M. smegmatis* ML16 strains expressing wt *mspA*, M1 *mspA*, M1-M1_19_ *mspA* were incubated with 1 µM ^14^C-labelled glucose at 37°C. Uptake of glucose by the porin triple deletion mutant of *M. smegmatis* ML16 carrying an empty vector pMS2 was very slow and was accelerated approximately 15-fold to wt levels by expressing either the *mspA* or M1-*mspA* gene (Fig. 6), consistent with previous reports [19]. These results showed that the aspartate to asparagine mutations in the constriction zone of MspA did not alter the permeability of the MspA pore to glucose *in vivo*. Importantly, expression of M1-M1_19_ *mspA* increased glucose uptake by *M. smegmatis* ML16 nine-fold demonstrating that the MspA pore made from dimers was also expressed in the outer membrane, retained its physiological function, and enabled diffusion of glucose *in vivo* (Fig. 6). It should be noted that the uptake of glucose mediated by M1-M1_19_ MspA was much higher than expected, considering the 12-fold reduced expression level of M1-M1_19_ MspA in ML16 compared to wt MspA (Fig. S2). This might be a result of a more efficient assembly of the MspA pore in the outer membrane of *M. smegmatis*.

### DNA Translocation and Nucleotide Recognition by the M1-M1_19_ Dimer Pore

**Figure 7:**
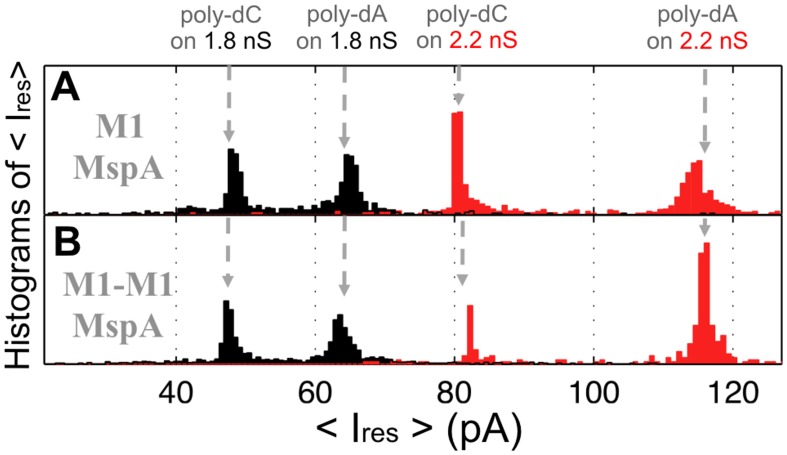
Histogram of the averaged residual ion current of single-stranded DNA homopolymers in M1 and M1-M1_19_ MspA Averaged Gaussian of I_res_ of M1 MspA (A) and M1-M1_19_ MspA (B) of ssDNA hairpins with homopolymeric poly-dA or poly-dC tails are shown. Data were recorded at 180 mV transmembrane potential. The data represent an average of four independent experiments.

The previous results did not reveal any difference between the MspA pores made from monomers or dimers. This means that formation of MspA pores by dimers could be utilized to improve the design of MspA for applications in nanotechnology. One of the most promising applications of MspA is nanosequencing of DNA [5], [11], [21]. To check whether the MspA pore made from M1-M1_19_ dimers is suitable for analyzing DNA, we examined interaction of the pore with single-stranded DNA hairpins. Previously we showed that M1 MspA was uniquely sensitive to nucleotides when double-stranded DNA arrested hairpin tail in the pore’s constriction [11]. In this experiment, upon addition of DNA to the grounded-chamber of the lipid bilayer and application of a positive voltage, hairpin DNA is drawn into the pore and stopped by the hairpin-duplex near protein constriction. Once lodged in the pore, the single-stranded nucleotides reduce the current to a residual value, I_res_, specific to the hairpin tail composition. After a few milliseconds the hairpin dissociates and the DNA fully translocates through the pore. While performing single pore bilayer experiments with M1 MspA, we observed several open-pore conductances (Fig. S6). We also observed a distinct association of the open-pore conductance with residual current, I_res_, caused by different hairpin-tail sequences. For example, M1 MspA with open conductance of 1.8 nS yielded I_res_ of ∼65 pA for a poly-dA tail; M1 MspA with open conductance of 2.2 nS yielded I_res_ of ∼115pA for the same poly-dA hairpin tail (Fig. 7, A). M1-M1_19_ MspA produced I_res_ values identical to that of M1 MspA with all hairpins tested in both 1.8 nS and 2.2 nS pores (Fig. 7, B). This result indicated that M1 MspA and M1-M1_19_ MspA interacted with ssDNA in a similar fashion suggesting that overall structure of both proteins is similar and that the MspA pore made from M1-M1_19_ dimers is suitable for DNA sequencing.

## Discussion

### A Wide Range of Peptide Linkers Enable Formation of Single-chain MspA Dimers in M. smegmatis

The octameric structure of the MspA-like porins is a novel feature of channel proteins and could be exploited to assemble channels of different stoichiometries. One approach to force formation of MspA pores by a different number of oligomers is to express genes encoding linked subunits. This approach has recently been adopted for the heptameric protein channel α-hemolysin [16]. However, it was unclear whether the MspA structure and a putative exposure of both termini of the mature protein to the outer membrane of *M. smegmatis* [17] are compatible with peptide linkers. In this study we provided the proof of concept that it is feasible to construct single-chain MspA dimers which assemble to channels indistinguishable from those formed by their monomeric counterparts. Surprisingly, peptide linkers with widely different lengths ranging from 17 to 62 amino acids enabled dimer formation in *M. smegmatis* (Fig. 2). Interestingly, the proportion of MspA pores made from monomers increased from 8% for the MspA-MspB_17_ construct to 48% for MspA-MspB_62_ indicating that longer linkers are prone to degradation (Fig. 2). A similar observation was made in *E. coli*: a poly-glycine linker connected two subunits in a chimeric protein, but was degraded by proteolytic enzymes and by a recombinant protease [28], [29]. Since MspA export is dependent on the Sec system in *M. smegmatis* [30], which transports unfolded proteins across the inner membrane [31], it is likely that linker degradation occurred in the periplasm by a yet unknown protease.

### Single-chain Dimers form Functional MspA Channels

In lipid bilayer experiments all Msp dimeric constructs showed channel forming activity (Fig. 3, 5, S4). If the gel-purified M1-M1_19_ sample had been contaminated with MspB we would have observed 2.4 nS and 3.4 nS peaks corresponding to MspB during bilayer experiments. However, M1-M1_19_ MspA and M1 MspA had identical conductance of 1.6 nS, indicating that at least the major fraction of the M1-M1_19_ sample consisted of oligomers made from dimers. This result was also confirmed by voltage gating experiments with M1-M1_19_ MspA and M1 MspA (Fig. S7). Voltage gaiting is a characteristic of β-barrel membrane channels [24]. Both proteins start to close at +40 mV in contrast to wt MspA [32]. These bilayer experiments demonstrated that all dimers formed functional channels in lipid bilayers, suggesting that neither sequence, nor length of the linker strongly influenced the overall structure, stoichiometry and channel properties of Msp pores made from dimers (Fig. 5). This is similar to results obtained with subunit dimers of α-hemolysin [16].

### Channels from Single-chain Dimers are Surprisingly Active in *M. smegmatis*

Glucose uptake experiments with whole cells demonstrated that the M1-M1_19_ MspA dimer was functionally expressed in the outer membrane of *M. smegmatis* (Fig. 6). M1-M1_19_ MspA enabled uptake of glucose by *M. smegmatis* with a rate of 50% of that of wt MspA and M1 MspA made from monomers despite a 12-fold reduced expression level. One explanation might be that M1-M1_19_ dimer forms more rigid oligomeric pores and has increased open state probability compared to wt MspA in *M. smegmatis* cells. Porins have traditionally been viewed as permanently open water filled channels that allow for passive diffusion of solutes [33]. However, this view has been challenged by studies which showed that the open state probability of the OmpF and OmpC pores in *E. coli* is regulated by pH, voltage and by small molecules which stabilize a closed pore conformation [34], [35], [36]. Although regulatory events of MspA channel activity are not known yet, it is likely that they exist considering the necessity of bacteria to adequately respond to rapid changes in environmental conditions such as osmolarity or presence of toxic solutes [37]. In this regard, it is tempting to speculate that the wt MspA pore might respond to these environmental signals by more or faster closing events than a pore made from subunit dimers. Obviously, further experiments are needed to test this hypothesis.

### Use of Single-chain MspA Dimers for Nanopore Sequencing of DNA

We compared M1-M1_19_ MspA subunit dimer with M1 MspA, a porin used for nanosequencing [11], [21]. Since the constriction zone is the same for both mutants, we expected that both constructs would interact similarly with DNA. Indeed, bilayer experiments revealed that the single-channel conductances observed for both proteins are very similar (Fig. S6). In addition, both proteins translocated ssDNA and produced identical I_res_ values for a given hairpin tail (Fig. 7). These results indicated that overall structure of both proteins is similar. More importantly, these experiments demonstrated that the pore made from M1-M1_19_ MspA subunit dimers is suitable for DNA sequencing. It should be noted that, regardless of the linker length and sequence, the expression levels of all constructs was approximately 10% of that of wt MspA (Fig. S2). This finding is consistent with the drop in yield for single-chain antibodies in *E. coli* [38]. Similarly, two- to five-fold lower expression levels of α-hemolysin from subunit dimers were observed [16]. Nevertheless, M1-M1_19_ MspA can be purified from *M. smegmatis* ML16 with a yield of 15 µg per liter of culture. This amount is sufficient for many applications including nanopore sequencing experiments. Approximately 2.5 ng/ml of M1 MspA is used for a single-pore experiment [11], [21]. Since the volume of the cuvette for experiments is 100 µl, 15 µg of the protein is enough to perform 60,000 experiments. If larger amounts of MspA protein are needed, other expression systems may be needed. For example, we have demonstrated that MspA can be expressed and purified from *E. coli* in mg quantities [7], [39].

### Conclusions

This study provides the proof of principle that production of single-chain MspA pores in *M. smegmatis* is feasible and paves the way for generating MspA pores with altered stoichiometries by using single-chain trimers or co-expression of genes encoding single chain trimers and tetramers. Such MspA channels enable us to probe the flexibility of MspA pore assembly and are likely to give rise to channels with altered constriction zone diameters. Utilization of subunit dimers reduces the number of mutations per pore and, thus, enables greater flexibility and better control of the chemical and physical properties of the constriction zone of MspA. This approach will be valuable both in biological experiments to better understand membrane permeability in mycobacteria and in tailoring MspA for nanotechnological applications such as nanopore sequencing of DNA.

## Materials and Methods

### Chemicals and Enzymes

Chemicals were of the highest purity available from Merck (Germany), Roth (Germany), Invitrogen (USA), or Sigma (USA) unless otherwise noted. The detergent *n*-octylpolyoxyethylene (n-octyl-POE) was from Alexis (USA). Oligonucleotides were obtained from Integrated DNA Technologies (USA).

### Bacterial Strains and Growth Conditions

*M. smegmatis* ML16, which lacks the porin genes *mspA*, *mspC*, and *mspD* [19] was grown at 37°C in 7H9 liquid medium (BD Biosciences) supplemented with 0.2% glycerol and 0.05% Tween 80 or on 7H10 agar (BD Biosciences) supplemented with 0.2% glycerol, unless otherwise indicated. *Escherichia coli* DH5α was used for cloning experiments and was routinely grown in Luria-Bertani broth (LB) at 37°C. Hygromycin was used in concentrations of 50 µg/ml and 200 µg/ml for *M. smegmatis* and *E*. *coli*, respectively.

### Construction of MspA Dimers

*E. coli* DH5α was used for construction of all plasmids (Table S1).

Plasmids pMN013, which carries a p_imyc_-*mspA* transcriptional fusion, and pMN042, which carries a p_smyc_-*mspB* transcriptional fusion, were used as parent plasmids for the construction of MspA-MspB dimers. First, pMN013 was used as a template to introduce EcoRV restriction site in front of the stop-codon of the *mspA* by site-directed mutagenesis using the combined chain reaction (CCR) [40]. The PCR fragment was digested with SphI and EcoRV, and ligated with appropriately digested pML972 to give pML869. Primer pMS-SEQ1 was used as reverse primer to amplify *mspB* from the plasmid pMN042 for the construction of different MspA-MspB dimers (Table S1). Different linkers, namely DI(GGGGS)_3,_ DI(GGGGS)_8,_ DI(GGGGS)_12,_ 38–51, 15–29, were introduced by the forward primers mspB-EcorV, mspB-EcorV-10, mspB-EcorV-6, L1, L2, respectively. The PCR fragments were digested by EcoRV and HindIII and ligated with appropriately digested pML869 to give pML870, pML870-10, pML870-6, pML871, pML872 (Table S1). Plasmid pML2610 which carries the *p_smyc_ - mspA M1 - mspA M1* translational fusion was used as a parent vector for the construction of pML2632. To this end, the primer M1 NsiI was used for mutagenesis by CCR to introduce the NsiI restriction site after the signal peptide encoding DNA sequence in the *mspA M1* gene. The CCR product was cloned into the vector pML2610 using the restriction enzymes NsiI and HindIII to yield pML2632 (Table S1). This plasmid carries a fusion of two *mspA M1* genes connected by a sequence encoding a (GGGGS)_3_ linker flanked by KpnI and NsiI restriction sites. Note, that the restriction sites added the amino acids GT and MH to the ends of the linker sequence (Table S1).

### Purification of MspA

MspA porins were selectively extracted from *M. smegmatis* and purified by subsequent anion exchange and gel filtration chromatography as described previously [10], [22].

### Analysis of MspA Mutants’ Expression by Gel Electrophoresis

To examine whether the MspA mutants were expressed in ML16, a selective extraction procedure of MspA was employed. *M. smegmatis* cells were heated in POP05 buffer (300 mM NaH_2_PO_4_/Na_2_HPO_4_, 0.3 mM Na_2_EDTA, 150 mM NaCl, 0.5% (w/v) n-octyl-POE) at a cell density of 100 µl/10 mg cells (wet weight) to 100°C for 30 min (2). Equal amounts of protein extracts were loaded and separated on a denaturing 10% polyacrylamide gel followed by staining with Coomassie Blue. Quantitative image analysis of protein gel bands by pixel densitometry was performed using Labworks 4.6 (UVP, Inc.) software.

### Denaturation of MspA

After a selective extraction of MspA as described above, the extracts were precipitated with ice-cold acetone and left on ice for 60 minutes followed by centrifugation for 15 minutes at 8000 g at 4°C. The pellet was resuspended in a buffer containing 0.5% (w/v) n-octyl-POE, 25 mM HEPES/NaOH, 10 mM NaCl, pH 7.5. The sample was dialyzed overnight at room temperature against PBS containing 0.5% (w/v) n-octyl-POE. Next, the sample was dried under vacuum and mixed with 125 µl of DMSO. 5µl of the sample were mixed with loading buffer and loaded onto 10% polyacrylamide gel followed by the transfer on PVDF membrane.

### Lipid Bilayer and Voltage Gating Experiments

The single-channel conductance of all MspA mutants was analyzed on a custom-made lipid bilayer apparatus as described previously [10]. Briefly, the Ag/AgCl electrodes were submerged in a solution of 1 M KCl, 10 mM HEPES, pH 7.0. The lipid membranes were painted from a solution of 1% diphytanoylphosphatidylcholine (DPhPC; Avanti Polar Lipids) in n-decane. Before adding the protein, current traces of at least three or more membranes were recorded with the 0.5% n-octyl-POE detergent in 25 mm sodium phosphate, pH 7.5 to exclude any contamination with channel forming activity and to demonstrate that the detergents did not affect the membrane. Then protein was added to both sides of the cuvette. Single-channel conductances for approximately 50 pores/sample were digitally recorded. Voltage was measured with a current amplifier (Keithley 428 current amplifier) and digitized by a desktop computer equipped with Keithley Metrabyte STA 1800 U interface. Data were recorded using a macro and the software Test Point 4.0 (Keithley). The raw data were analyzed using IGOR Pro 5.03 program (WaveMetrics) and a macro provided by Dr. Harald Engelhardt. These data were further analyzed in SigmaPlot 9.0 (Systat Software) to generate the figures shown here.

For voltage gating experiments the protein was added to the cuvette on the *cis* side which was defined as the side with the positive electrode. Upon insertion of approximately 100 pores per membrane the applied voltage was increased from 0 mV to −60 mV or +60 mV in 10 mV increments. The data were recorded and analyzed as described above.

### Synthesis and Structure of DNA Hairpins

DNA hairpins were synthesized by Integrated DNA Technologies (IDT). To prevent self-dimerization hairpins were heated at 90°C for 1 minute, cooled in a −20°C freezer for an additional minute, and then equilibrated to room temperature before use. DNA was added to the grounded chamber at a final concentration of about 10 µM. Hairpin DNA sequences had a 14 base duplex region and 6 nt loop: 5′-GCTGGCTCTGTTGC TCTCTC GCAACAGAGCCAGC <tail>-3′. The underlined sequences indicate duplex formation between complementary bases and the hairpin tail sequences were (dA)_60_ or (dC)_50_ homopolymers.

### Single-pore DNA Experiments

In single-pore DNA translocation experiments the pores were established with previously described methods [21]. Briefly, lipid bilayers were formed from 1,2-diphytanoyl-sn-glycerol-3-phosphocholine (Avanti Polar Lipids). The bilayer spanned a horizontal ∼20 µm diameter aperture in Teflon. M1 MspA or M1-M1_19_ MspA was added to the grounded side of the bilayer at a concentration of ∼2.5 ng/ml. An Axopatch-1B or 200B patch clamp amplifier (Axon Instruments) applied a voltage across the bilayer and measured the ionic currents. The analog signal was low-pass filtered at 10, 50 or 100 kHz with a 4-pole Bessel filter and was then digitized at five times the low-pass filter frequency. Data acquisition was controlled with custom software written in LabWindows/CVI (National Instruments). All experiments were performed at 23±1°C in 1 M KCl, 10 mM HEPES/KOH, pH 8.0 with expected small changes in salinity due to evaporation. The data were analyzed with custom software written in Matlab (The Mathworks).

### Glucose Uptake Measurements

Glucose uptake experiments were carried out as previously described [19] with minor modifications. Briefly, the cells were grown at 37°C in Middlebrook 7H9 medium supplemented with 0.01% Tyloxapol and 200 µg/ml of hygromycin. The 100 ml cell cultures were centrifuged at 3250 g at 4°C for 10 min at an OD_600_ of 0.6, washed twice in the uptake buffer (50 mM Tris-HCl, 15 mM KCl, 10 mM (NH4)_2_SO_4_, 0.01% Tyloxapol, pH 6.9) and resuspended in the same buffer. Radiolabelled [^14^C]glucose (GE healthcare) was added to cell suspensions to obtain final concentrations of 1 µM. The mixtures were incubated at 37°C and 0.2 ml of samples were removed at the indicated times. The cells were collected on a 0.45 mm Spin-X centrifuge tube filter (Costar) by mixing with an equal volume of 10% buffered formalin phosphate (Fisher) containing 0.1 M LiCl, washed twice in the same buffer, and counted in a liquid scintillation counter (Beckman). Uptake rates were expressed as nmol/mg cells (dry weight).

### Gel-extraction of MspA-MspB_17_ and M1-M1_19_ MspA Oligomeric Bands

To obtain pure MspA-MspB_17_ and M1-M1_19_ MspA oligomers we first extracted the proteins from *M. smegmatis* ML 16 by boiling the cells in a buffer containing 0.5% n-octyl-POE as described above. Then, the extract was concentrated using Amicon filter tubes (Millipore) with a molecular weight cut-off of 3 kDa. The retentate was loaded onto an SDS-polyacrylamide gel followed by staining with Simple Blue Safe Stain (Invitrogen). The band corresponding to the octamer made from subunit dimers was cut from the gel and electro-eluted. Next, the sample was dialyzed overnight at room temperature against PBS containing 0.5% (w/v) n-octyl-POE. After dialysis the sample was analyzed for channel forming activity as described above.

## Supporting Information

Figure S1 **Denaturation of MspA-MspB dimers.** After selective extraction of MspA-MspB dimers from *M. smegmatis* ML16 at 100°C, proteins were denatured with DMSO. Approximately 5 µg of the protein was loaded onto 10% polyacrylamide gel followed by the transfer on PVDF membrane. The Western blot was probed with MspA antiserum. Lanes: 1, denatured wt MspA; 2, extract from *M. smegmatis* ML16 containing the empty vector pMS2; 3, MspA-MspB_17_; 4, MspA-MspB_42_; 5, MspA-MspB_62_; 6, MspA-MspB_16LTR_; 7, MspA-MspB_14TLT_. Abbreviations: d, dimeric form; m, monomeric form. For mutants description see Table 1 in the main text.
(TIF)

Figure S2 **Expression levels of MspA dimers with different linkers.** (A) Analysis of the expression levels of MspA different MspA dimers by gel electrophoresis. After selective extraction of MspA proteins at 100°C from *M. smegmatis* ML16 cells 20 µl of the extract have been loaded onto 10% polyacrylamide gel followed by staining with Coomassie Blue. *Top panel*. Lanes: M, molecular weight marker EZ-Run Pre-stained Rec Protein Ladder (Fisher); lane 1, wt MspA; lane 2, extract from *M. smegmatis* ML16 bearing empty vector pMS2; lane 3, MspA-MspB_17_; lane 4, MspA-MspB_42_; lane 5, MspA-MspB_62_; lane 6, MspA-MspB_16LTR_; lane 7, MspA-MspB_14TLT_. For mutants description see Table 1 in the main text. *Bottom panel*. Lanes: M, molecular weight marker EZ-Run Pre-stained Rec Protein Ladder (Fisher); lane 1, wt MspA; lane 2, extract from *M. smegmatis* ML16 bearing empty vector pMS2; lane 3, M1 MspA; lane 4, M1-M1_19_ MspA dimer. For mutants description see Table 1 in the main text. (B) Quantitative image analysis of the gel bands shown in A. Image analysis of protein gel bands by pixel densitometry was performed using Labworks 4.6 (UVP, Inc.) software. Data are represented as the percentage of the wt MspA expression. Bars represent pixel densitometry of the corresponding bands from panel A. Bars: 1, wt MspA; 2, extract from *M. smegmatis* ML16 bearing empty vector pMS2; 3, M1 MspA; 4, M1-M1_19_ MspA; 5, MspA-MspB_17_; 6, MspA-MspB_42_; 7, MspA-MspB_62_; 8, MspA-MspB_16LTR_; 9, MspA-MspB_14TLT_.
(TIF)

Figure S3 **Characterization of MspA and MspB in lipid bilayers.** Single channel recordings of purified MspA (A) and MspB (C) in a diphytanoyl phosphatidylcholine (DphPC) membrane in the presence of approximately 100 ng/mL protein sample. Protein solutions were added to both sides of the membrane and data were collected from at least four different membranes. −10 mV transmembrane potential was applied and current was measured in 1 M KCl solution, pH 7.0 Analysis of single channel conductances of MspA (B) and MspB (D). Analysis of the probability P of a conductance step G for single channel events. The average single channel conductances were 4.9 nS for MspA, and 2.3 nS and 3.4 nS for MspB.
(TIF)

Figure S4 **Characterization of MspA-MspB dimers in lipid bilayers.** Single channel recordings of purified in a diphytanoyl phosphatidylcholine (DphPC) membrane in the presence of approximately 100 ng/mL protein sample. Protein solutions were added to both sides of the membrane and data were collected from at least four different membranes. −10 mV transmembrane potential was applied and current was measured in 1 M KCl solution, pH 7.0. Analysis of single channel conductances of MspA (B), MspA-MspB_42_ (D), MspA-MspB_62_ (F), MspA-MspB_16LTR_ (H), and MspA-MspB_14TLT_ (J). Data are expressed as the probability P of a conductance step G for single channel events. The average single channel conductances were 4.9 nS for MspA, and 2.4 nS for MspA-MspB_42_, MspA-MspB_62_, and MspA-MspB_16LTR_. Analysis of MspA-MspB_14TLT_ showed multiple peaks at 0.9 nS, 2.4 nS, and 4.9 nS.
(TIF)

Figure S5 **Purification of the M1-M1_19_ MspA dimer from the porin mutant** ***M. smegmatis*** **ML16.** (A) Anion exchange chromatography of M1-M1_19_ dimer. The solid line represents the absorbance at 280 nm. Linear gradient from 0 to 2 M NaCl eluted M1-M1_19_ MspA at 0.6 M NaCl. (B). Analysis of anion exchange fractions by gel electrophoresis. The fractions (#12–21) were loaded into 8% SDS-PAGE and stained with silver nitrate. (C) Gel electrophoresis analysis of the purification steps. Proteins were separated in 8% SDS-PAGE and stained with silver nitrate. 15 µl of sample after each purification step was loaded on the gel. Lane 1, sample after the POP05 extraction; lane 2, protein of the sample of lane 1 after precipitation with acetone; lane 3, sample from fraction #16 of the anion exchange run as in (B); lane 4, sample of lane 3 after dialysis against PBS, 0.5% OPOE, pH 7.4; lane 5, flow through of the concentration step with Amicon filters (MWCO 12 kDa); lane 6, sample after concentration with Amicon filters (MWCO 12 kDa). The final concentration of the purified M1-M1_19_ MspA was 0.2 µg/ml as determined by bicinchoninic acid.
(TIF)

Figure S6 **Distribution of conductances of M1 MspA and M1-M1_19_ MspA in a single pore bilayer experiments.** Lipid bilayers were formed from 1,2-diphytanoyl-sn-glycerol-3-phosphocholine (Avanti Polar Lipids). The bilayer spanned a horizontal ∼20 µm diameter aperture in Teflon. M1 MspA or M1-M1_19_ MspA was added to the grounded side of the bilayer at a concentration of ∼2.5 ng/ml. An Axopatch-1B or 200B patch clamp amplifier (Axon Instruments) applied a voltage across the bilayer and measured the ionic currents. All experiments were performed at 23±1°C in 1 M KCl, 10 mM HEPES/KOH, pH 8.0 with expected small changes in salinity due to evaporation. The data were analyzed with custom software written in Matlab (The Mathworks). Data are expressed as the frequency of the conductivity of a particular single channel event.
(TIF)

Figure S7 **Voltage gating of M1 MspA and M1-M1_19_ MspA.** Purified MspA was added to the *cis*-side of a DphPC membrane. Increasingly positive (upper traces) and negative (lower traces) voltages were applied to the membrane when ∼100 channels were reconstituted into the membrane. The membrane current was recorded at each applied voltage. The critical voltage at which the channels began to close (*V_c_*) was determined to be the voltage where conductance decreased after an initial spike. Both M1 MspA (A) and M1-M1_19_ MspA (B) were measured to have a *Vc* of ±50 mV.
(TIF)

Table S1 **Strains, plasmids and oligonucleotides used in this work.** The annotation Hyg^R^ indicates resistance to hygromycin. *MspA, mspC, mspD* are porin genes of *M. smegmatis*. The codons that were altered to introduce the MspA mutations are underlined.
(DOCX)
