# Supplementary figures and images for: MspA Nanopores from Subunit Dimers
Source: PLoS One. 2012 Jun 18;7(6):e38726. doi: 10.1371/journal.pone.0038726 (PMC3377714; doi:10.1371/journal.pone.0038726)

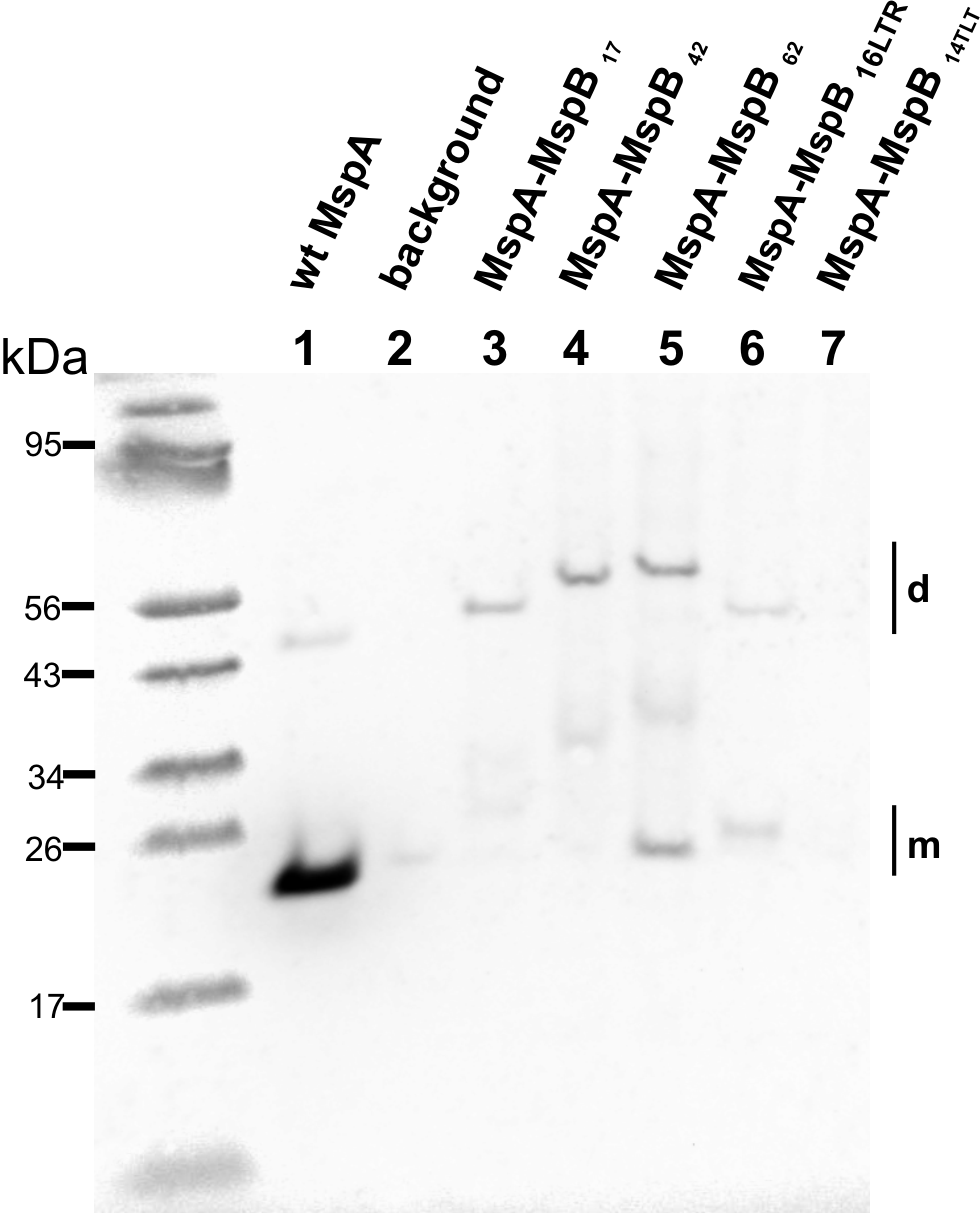

Supplement: Figure S1 — Denaturation of MspA-MspB dimers. After selective extraction of MspA-MspB dimers from M. smegmatis ML16 at 100°C, proteins were denatured with DMSO. Approximately 5 µg of the protein was loaded onto 10% polyacrylamide gel followed by the transfer on PVDF membrane. The Western blot was probed with MspA antiserum. Lanes: 1, denatured wt MspA; 2, extract from M. smegmatis ML16 containing the empty vector pMS2; 3, MspA-MspB17; 4, MspA-MspB42; 5, MspA-MspB62; 6, MspA-MspB16LTR; 7, MspA-MspB14TLT. Abbreviations: d, dimeric form; m, monomeric form. For mutants description see Table 1 in the main text. (TIF) [file pone.0038726.s001.tif]

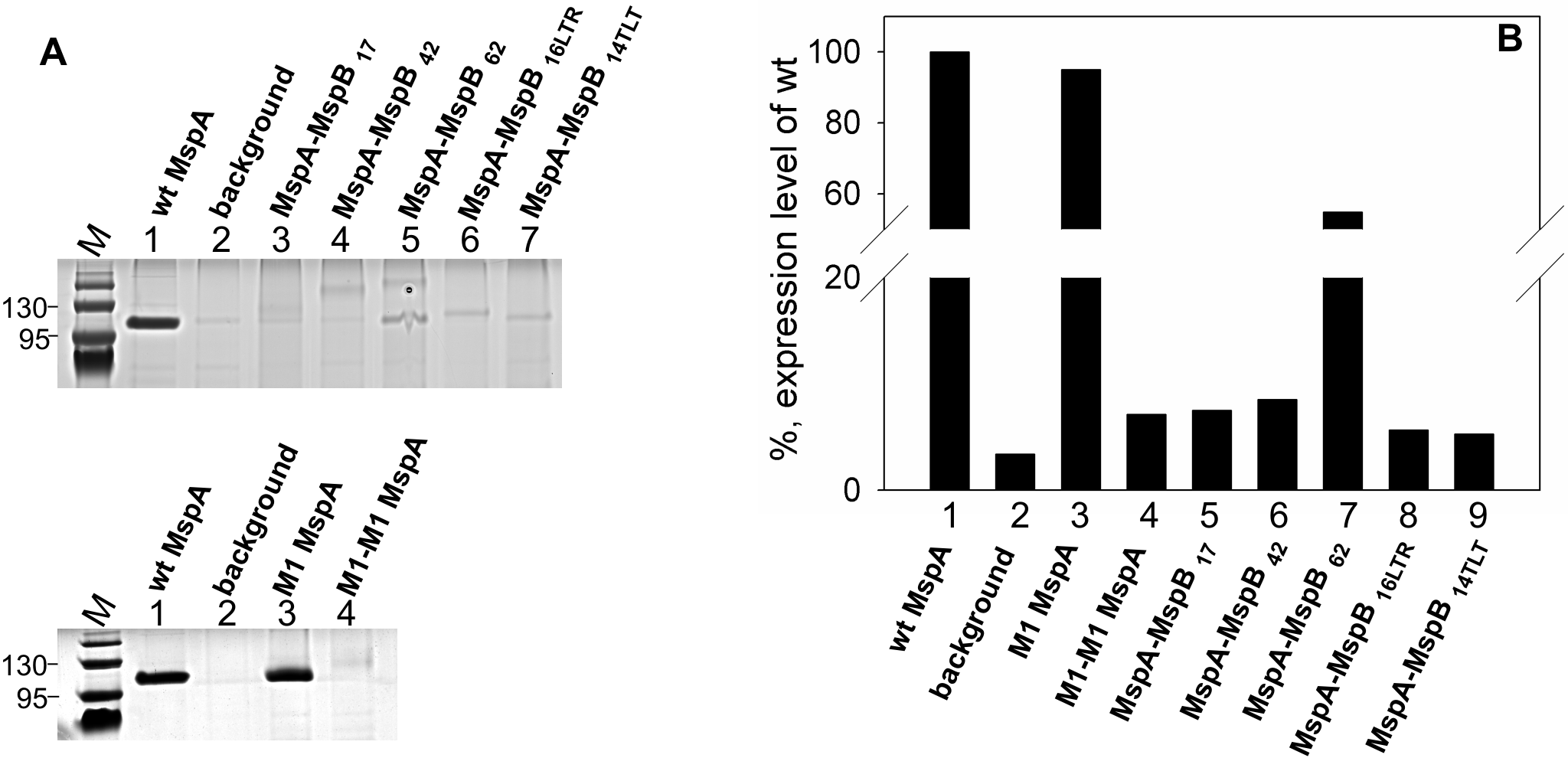

Supplement: Figure S2 — Expression levels of MspA dimers with different linkers. (A) Analysis of the expression levels of MspA different MspA dimers by gel electrophoresis. After selective extraction of MspA proteins at 100°C from M. smegmatis ML16 cells 20 µl of the extract have been loaded onto 10% polyacrylamide gel followed by staining with Coomassie Blue. Top panel. Lanes: M, molecular weight marker EZ-Run Pre-stained Rec Protein Ladder (Fisher); lane 1, wt MspA; lane 2, extract from M. smegmatis ML16 bearing empty vector pMS2; lane 3, MspA-MspB17; lane 4, MspA-MspB42; lane 5, MspA-MspB62; lane 6, MspA-MspB16LTR; lane 7, MspA-MspB14TLT. For mutants description see Table 1 in the main text. Bottom panel. Lanes: M, molecular weight marker EZ-Run Pre-stained Rec Protein Ladder (Fisher); lane 1, wt MspA; lane 2, extract from M. smegmatis ML16 bearing empty vector pMS2; lane 3, M1 MspA; lane 4, M1-M119 MspA dimer. For mutants description see Table 1 in the main text. (B) Quantitative image analysis of the gel bands shown in A. Image analysis of protein gel bands by pixel densitometry was performed using Labworks 4.6 (UVP, Inc.) software. Data are represented as the percentage of the wt MspA expression. Bars represent pixel densitometry of the corresponding bands from panel A. Bars: 1, wt MspA; 2, extract from M. smegmatis ML16 bearing empty vector pMS2; 3, M1 MspA; 4, M1-M119 MspA; 5, MspA-MspB17; 6, MspA-MspB42; 7, MspA-MspB62; 8, MspA-MspB16LTR; 9, MspA-MspB14TLT. (TIF) [file pone.0038726.s002.tif]

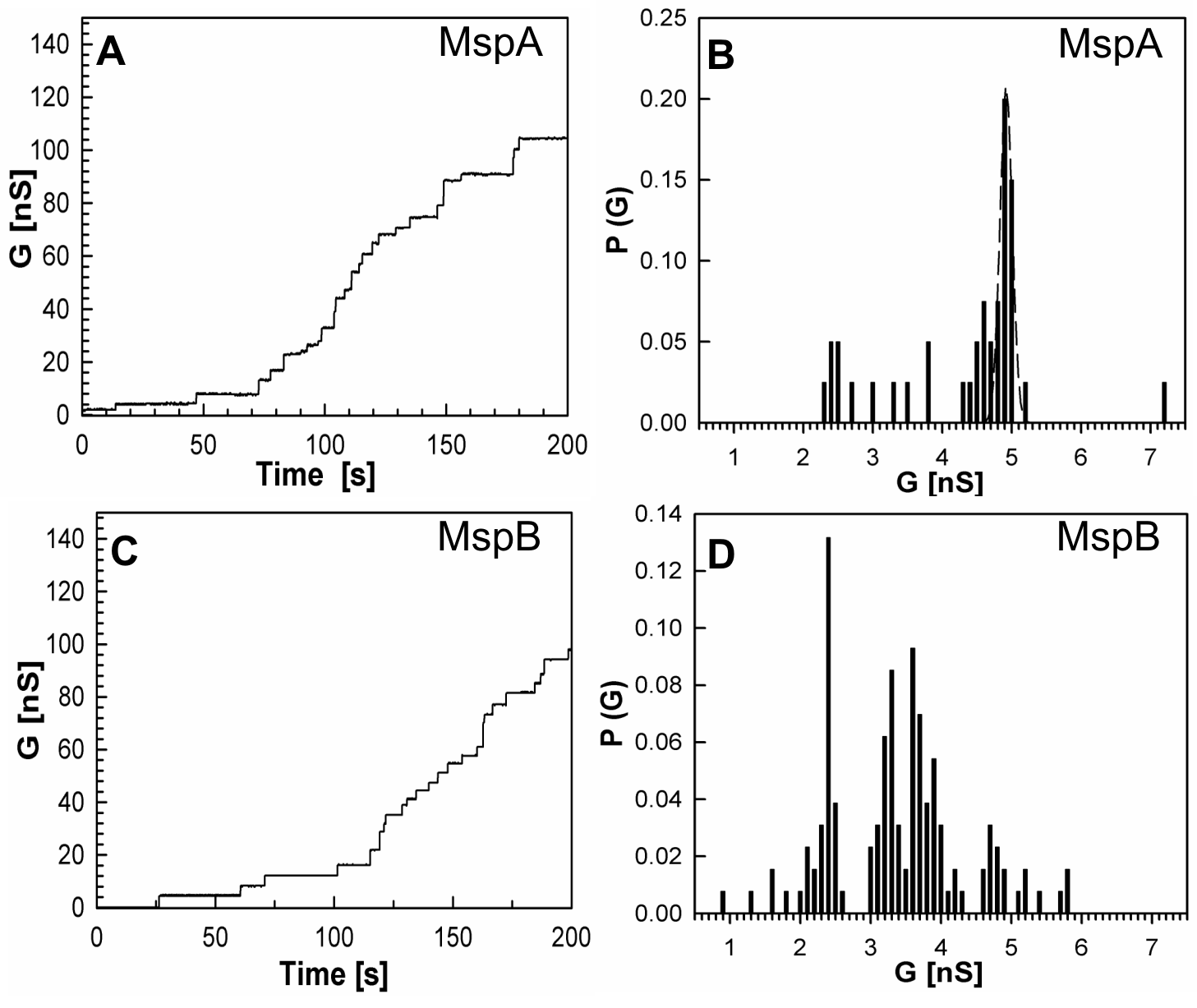

Supplement: Figure S3 — Characterization of MspA and MspB in lipid bilayers. Single channel recordings of purified MspA (A) and MspB (C) in a diphytanoyl phosphatidylcholine (DphPC) membrane in the presence of approximately 100 ng/mL protein sample. Protein solutions were added to both sides of the membrane and data were collected from at least four different membranes. −10 mV transmembrane potential was applied and current was measured in 1 M KCl solution, pH 7.0 Analysis of single channel conductances of MspA (B) and MspB (D). Analysis of the probability P of a conductance step G for single channel events. The average single channel conductances were 4.9 nS for MspA, and 2.3 nS and 3.4 nS for MspB. (TIF) [file pone.0038726.s003.tif]

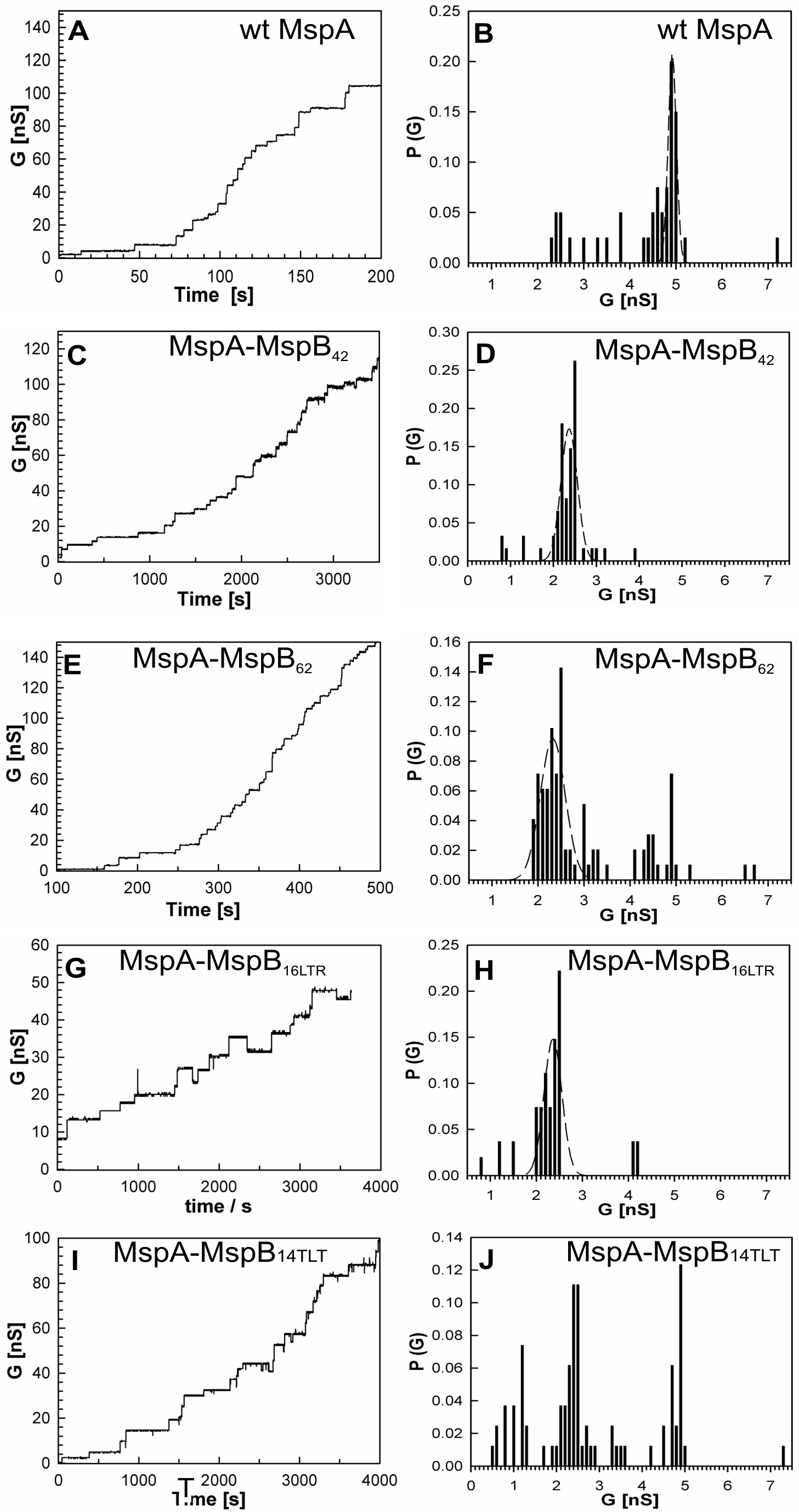

Supplement: Figure S4 — Characterization of MspA-MspB dimers in lipid bilayers. Single channel recordings of purified in a diphytanoyl phosphatidylcholine (DphPC) membrane in the presence of approximately 100 ng/mL protein sample. Protein solutions were added to both sides of the membrane and data were collected from at least four different membranes. −10 mV transmembrane potential was applied and current was measured in 1 M KCl solution, pH 7.0. Analysis of single channel conductances of MspA (B), MspA-MspB42 (D), MspA-MspB62 (F), MspA-MspB16LTR (H), and MspA-MspB14TLT (J). Data are expressed as the probability P of a conductance step G for single channel events. The average single channel conductances were 4.9 nS for MspA, and 2.4 nS for MspA-MspB42, MspA-MspB62, and MspA-MspB16LTR. Analysis of MspA-MspB14TLT showed multiple peaks at 0.9 nS, 2.4 nS, and 4.9 nS. (TIF) [file pone.0038726.s004.tif]

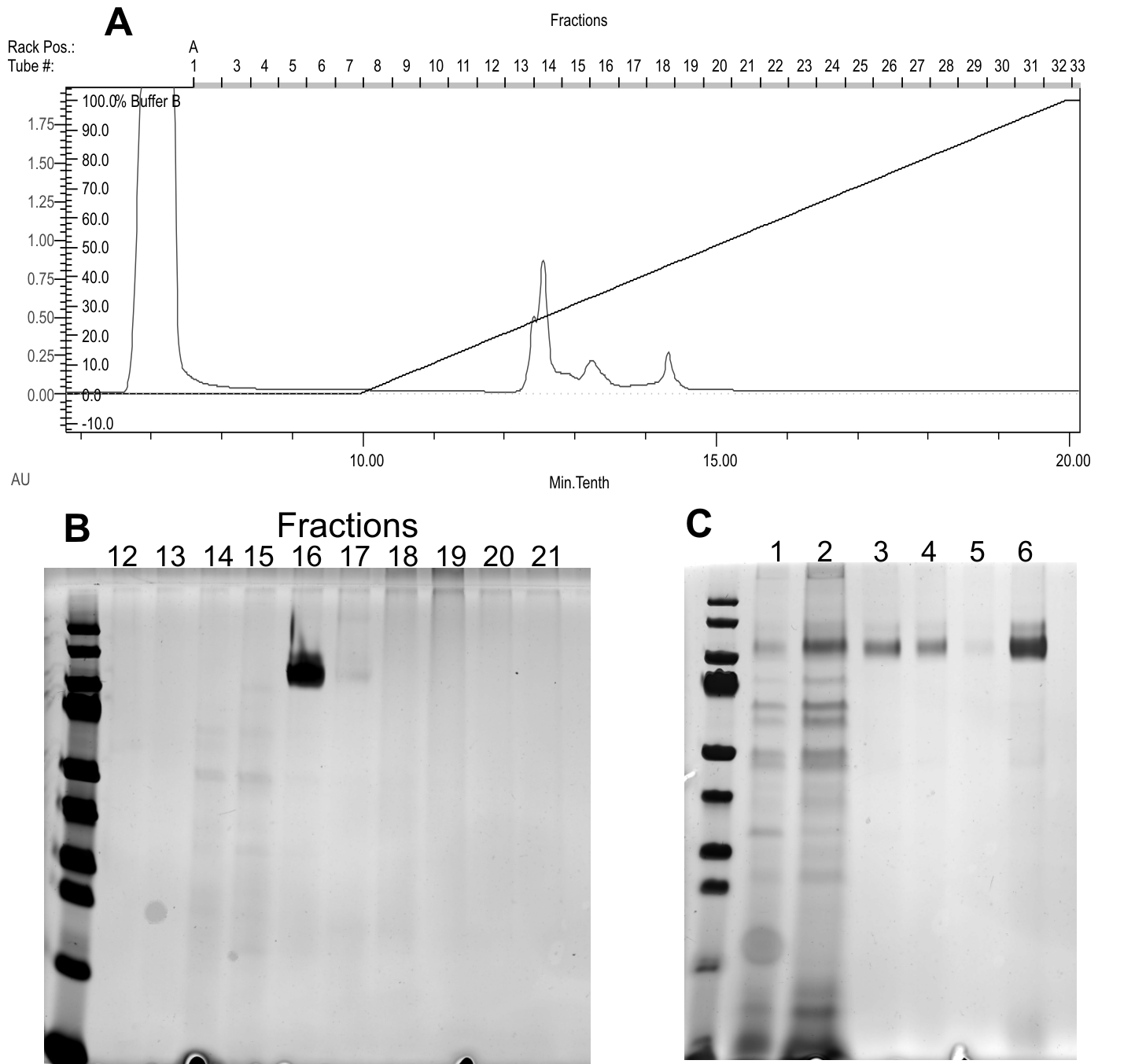

Supplement: Figure S5 — Purification of the M1-M119 MspA dimer from the porin mutant M. smegmatis ML16. (A) Anion exchange chromatography of M1-M119 dimer. The solid line represents the absorbance at 280 nm. Linear gradient from 0 to 2 M NaCl eluted M1-M119 MspA at 0.6 M NaCl. (B). Analysis of anion exchange fractions by gel electrophoresis. The fractions (#12–21) were loaded into 8% SDS-PAGE and stained with silver nitrate. (C) Gel electrophoresis analysis of the purification steps. Proteins were separated in 8% SDS-PAGE and stained with silver nitrate. 15 µl of sample after each purification step was loaded on the gel. Lane 1, sample after the POP05 extraction; lane 2, protein of the sample of lane 1 after precipitation with acetone; lane 3, sample from fraction #16 of the anion exchange run as in (B); lane 4, sample of lane 3 after dialysis against PBS, 0.5% OPOE, pH 7.4; lane 5, flow through of the concentration step with Amicon filters (MWCO 12 kDa); lane 6, sample after concentration with Amicon filters (MWCO 12 kDa). The final concentration of the purified M1-M119 MspA was 0.2 µg/ml as determined by bicinchoninic acid. (TIF) [file pone.0038726.s005.tif]

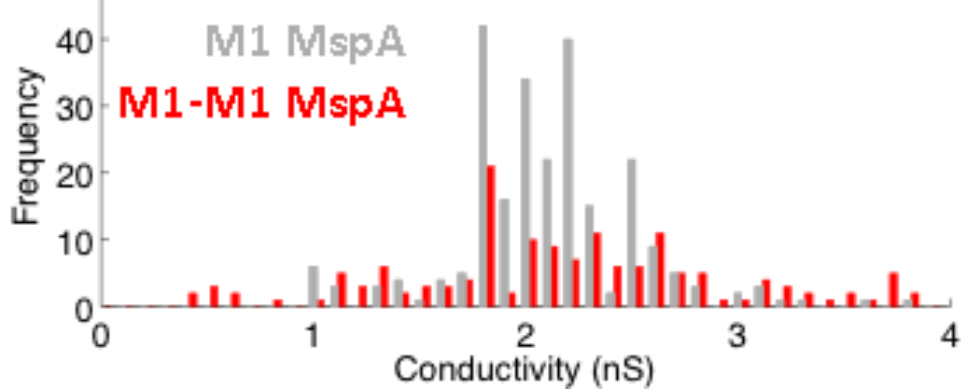

Supplement: Figure S6 — Distribution of conductances of M1 MspA and M1-M119 MspA in a single pore bilayer experiments. Lipid bilayers were formed from 1,2-diphytanoyl-sn-glycerol-3-phosphocholine (Avanti Polar Lipids). The bilayer spanned a horizontal ∼20 µm diameter aperture in Teflon. M1 MspA or M1-M119 MspA was added to the grounded side of the bilayer at a concentration of ∼2.5 ng/ml. An Axopatch-1B or 200B patch clamp amplifier (Axon Instruments) applied a voltage across the bilayer and measured the ionic currents. All experiments were performed at 23±1°C in 1 M KCl, 10 mM HEPES/KOH, pH 8.0 with expected small changes in salinity due to evaporation. The data were analyzed with custom software written in Matlab (The Mathworks). Data are expressed as the frequency of the conductivity of a particular single channel event. (TIF) [file pone.0038726.s006.tif]

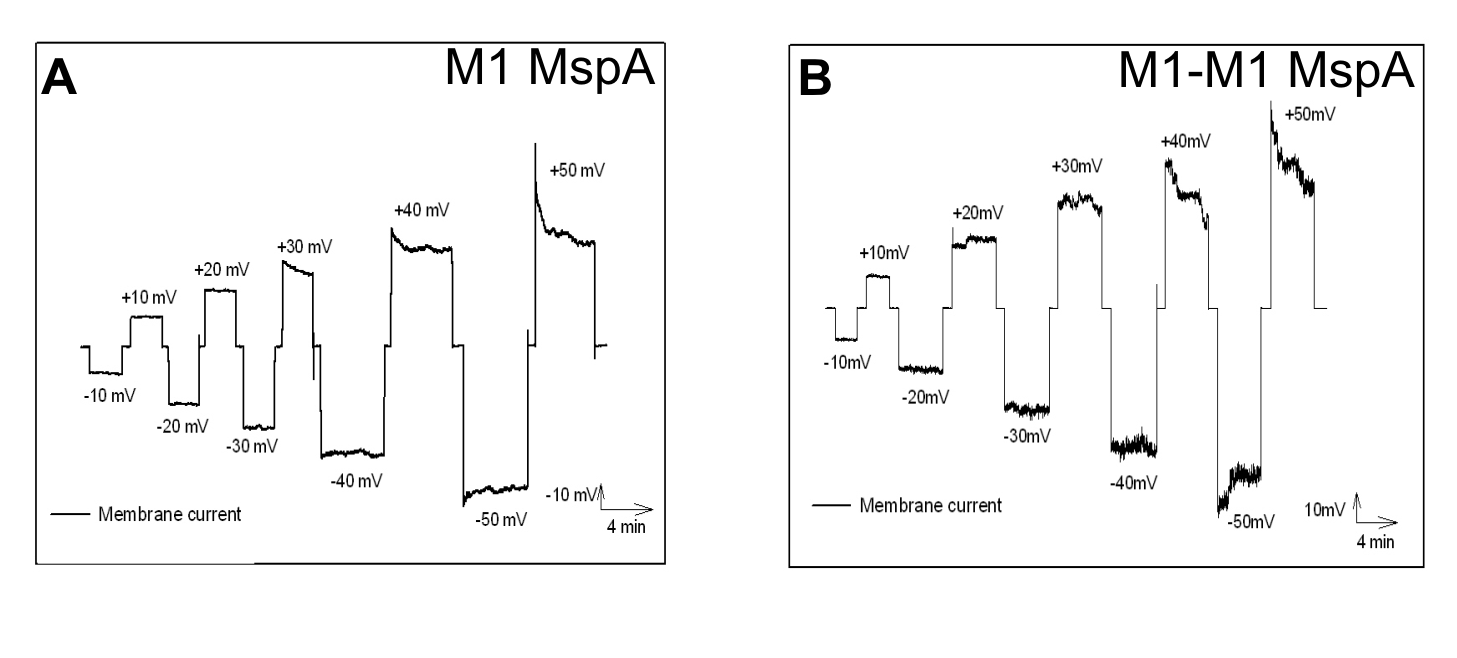

Supplement: Figure S7 — Voltage gating of M1 MspA and M1-M119 MspA. Purified MspA was added to the cis-side of a DphPC membrane. Increasingly positive (upper traces) and negative (lower traces) voltages were applied to the membrane when ∼100 channels were reconstituted into the membrane. The membrane current was recorded at each applied voltage. The critical voltage at which the channels began to close (Vc) was determined to be the voltage where conductance decreased after an initial spike. Both M1 MspA (A) and M1-M119 MspA (B) were measured to have a Vc of ±50 mV. (TIF) [file pone.0038726.s007.tif]
