# Supplementary material for: MspA Nanopores from Subunit Dimers
Source: PLoS One. 2012 Jun 18;7(6):e38726. doi: 10.1371/journal.pone.0038726 (PMC3377714; doi:10.1371/journal.pone.0038726)
Supplement: Table S1 — Strains, plasmids and oligonucleotides used in this work. The annotation HygR indicates resistance to hygromycin. MspA, mspC, mspD are porin genes of M. smegmatis. The codons that were altered to introduce the MspA mutations are underlined. (DOCX) [file pone.0038726.s008.docx]

| **Strain** | **Parent strain and relevant genotype** | **Source, reference, notes** | |
| --- | --- | --- | --- |
| *E. coli* DH5α | *rec*A1, *end*A1, *gyr*A96, *thi*; *rel*A1,*hsd*R17(r_K_^-^,m_K_^+^), *sup*E44, φ80Δ*lacZ*ΔM15, Δ*lacZ*(YA-argF)UE169 | (1) | |
| *M. smegmatis* ML16 | ML15, Δ*mspA*::*FRT*, Δ*mspC*::*FRT*, Δ*mspD*::*FRT, attB::loxP, FRT*, Hyg^R^ | (2) | |
|  |  |  | |
| **Plasmid** | **Parent vector, relevant genotype and properties** |  | |
| pMS2 | ColE1 origin, PAL5000 origin, Hyg^R^ | (3) | |
| pMN016 | p_smyc_-*mspA,* ColE1 origin, PAL5000 origin, Hyg^R^ | (2) | |
| pML904 | pMN016 derivative, *p_smyc_-mspA D909193N* | (4) | |
| pMN042 | p_smyc_-*mspB,* ColE1 origin, PAL5000 origin, Hyg^R^ |  | |
| pMN013 | p_imyc_-*mspA,* ColE1 origin, PAL5000 origin, Hyg^R^ |  | |
| pML972 | p_smyc_-*msmeg3748,*MCS, ColE1 origin, PAL5000 origin, Hyg^R^ |  | |
| pML869 | pML972 derivative, *p_smyc_-mspA(EcoRV),* ColE1 origin, PAL5000 origin, Hyg^R^ | this study | |
| pML2610 | pMN016 derivative, *p_smyc_-mspAM1-mspA1*,ColE1 origin, PAL5000 origin, Hyg^R^ | this study | |
| pML870 | pML869 derivative, *p_smyc_ -mspA-mspB*_17_ *,* ColE1 origin, PAL5000 origin, Hyg^R^ | this study | |
| pML870-10 | pML869 derivative, *p_smyc_ -mspA-mspB*_42_ *,* ColE1 origin, PAL5000 origin, Hyg^R^ *_c_* | this study | |
| pML870-6 | pML869 derivative, *p_smyc_ -mspA-mspB*_62_ *,* ColE1 origin, PAL5000 origin, Hyg^R^ | this study | |
| pML871 | pML869 derivative, *p_smyc_ -mspA-mspB*_16LTR_ *,* ColE1 origin, PAL5000 origin, Hyg^R^ | this study | |
| pML872 | pML869 derivative, *p_smyc_ -mspA-mspB*_14TLT_ *,* ColE1 origin, PAL5000 origin, Hyg^R^ | this study | |
| pML2632 | pML2610 derivative, *p_smyc_ –mspAM1-mspAM1* *,* ColE1 origin, PAL5000 origin, Hyg^R^ | this study | |
|  |  |  | |
| **Oligonucleotide** | **Sequence (5’ to 3’ direction)** |  | |
| COLE1 | GCGAGTCAGTGAGCGAGGAAGCG | | Amplification and sequencing of *msp*A |
| pMS-SEQ1 | CGTTCTCGGCTCGATGATCC | | Amplification and sequencing of *msp*A |
| mspA_EcoRV | GCGATATCGTTCATGTTCCAGGGTTCGCCG | | Cloning of pML870 |
| mspB_EcoRV | CGGATATCGGCGGTGGCGGTAGCGGCGGTGGCGGTAGCGGCGGTGGCGGTAGCGGGCTCGACAACGAATTGAGC | | Cloning of pML870 |
| M1 NsiI | AAAAAAAAAATGCATGGCCTGGACAACGAGCTGAGCCTCGTTGAT | | Cloning of pML2632 |

**References**

1. Hanahan, D. (1983) *J Mol Biol* **166**, 557-580

2. Stephan, J., Bender, J., Wolschendorf, F., Hoffmann, C., Roth, E., Mailänder, C., Engelhardt, H., and Niederweis, M. (2005) *Mol. Microbiol.* **58**, 714-730

3. Kaps, I., Ehrt, S., Seeber, S., Schnappinger, D., Martin, C., Riley, L. W., and Niederweis, M. (2001) *Gene* **278**, 115-124

4. Butler TZ, Pavlenok M, Derrington IM, Niederweis M, Gundlach JH. (2008) Proc Natl Acad Sci U S A;105(52):20647-52
